# Therapeutic Plasma Exchange in Immune-Mediated Thrombotic Thrombocytopenic Purpura: From Cornerstone to Contextualized Therapy

**DOI:** 10.3390/jcm15176683

**Published:** 2026-08-28

**Authors:** Fedai Özcan, Alexandra Brinkhoff, Ralph Wendt

**Affiliations:** 1 Department of Nephrology, Klinikum Dortmund, 44137 Dortmund, Germany; alexandra.brinkhoff@klinikumdo.de; 2 Faculty of Medicine, University of Witten/Herdecke, 58455 Witten, Germany; 3 Department of Nephrology, Klinikum St. Georg, 04129 Leipzig, Germany; ralph.wendt@sanktgeorg.de

**Keywords:** immune-mediated thrombotic thrombocytopenic purpura, caplacizumab, therapeutic plasma exchange, immunosuppression, ADAMTS13

## Abstract

Immune-mediated thrombotic thrombocytopenic purpura (iTTP) is a rare, life-threatening thrombotic microangiopathy caused by severe ADAMTS13 deficiency due to anti-ADAMTS13 autoantibodies. The resulting persistence of ultra-large von Willebrand factor (VWF) multimers promotes uncontrolled platelet adhesion and aggregation in the microcirculation, leading to thrombocytopenia, microangiopathic hemolytic anemia, and ischemic organ injury. Therapeutic plasma exchange (TPE) has transformed the prognosis of iTTP by removing circulating autoantibodies and replenishing functional ADAMTS13, and it remains a life-saving intervention in acute disease. However, TPE is invasive, resource-intensive, dependent on central venous access and plasma availability, and associated with catheter-related, hemodynamic, metabolic, infectious, and plasma-related adverse events. The therapeutic landscape has changed substantially with the incorporation of immunosuppression and the anti-VWF nanobody caplacizumab. Caplacizumab rapidly blocks VWF–platelet interactions at the effector level, whereas corticosteroids and B-cell-directed therapy target the autoimmune basis of iTTP. Triple therapy with TPE, immunosuppression, and caplacizumab accelerates platelet recovery and reduces unfavorable outcomes. At the same time, accumulating observational evidence and early prospective data suggest that selected patients may achieve remission with caplacizumab plus immunosuppression without routine first-line TPE. This perspective review critically re-evaluates the role of TPE in contemporary iTTP management. We propose that TPE should no longer be viewed exclusively as an obligatory universal first-line intervention, but rather as a contextualized component of individualized, response-adapted care. TPE remains indispensable for severe, unstable, or refractory disease and must be immediately available when TPE-free treatment is attempted. Safe implementation of TPE-free strategies requires experienced centers, rapid ADAMTS13 testing, immediate access to caplacizumab and immunosuppression, careful patient selection, and close clinical and laboratory monitoring. Defining which patients can be treated safely without TPE is a central challenge for future trials and guideline development.

## 1. Introduction

Thrombotic thrombocytopenic purpura (TTP) is a rare but life-threatening thrombotic microangiopathy characterized by severe thrombocytopenia, microangiopathic hemolytic anemia, and ischemic organ injury caused by disseminated microvascular platelet-rich thrombi [1]. Without effective treatment, the disease is fatal in most patients [1]. The majority of cases are immune-mediated (iTTP) and result from autoantibodies that cause severe deficiency of ADAMTS13, a plasma metalloprotease that cleaves ultra-large VWF multimers [1]. Severe ADAMTS13 deficiency allows highly adhesive VWF multimers to persist, thereby promoting platelet adhesion, aggregation, and microvascular occlusion [2,3].

The introduction of therapeutic plasma exchange (TPE) in the early 1990s fundamentally changed the prognosis of iTTP, increasing survival to approximately 85% [4]. TPE removes circulating anti-ADAMTS13 autoantibodies, supplies functional ADAMTS13, and reduces ultra-large VWF multimers and immune complexes, thereby interrupting the pathophysiological cascade that drives acute microvascular thrombosis [5]. These effects established TPE as the central acute intervention in iTTP and have shaped clinical practice and guideline recommendations for decades [5,6,7,8].

**Figure 1:**
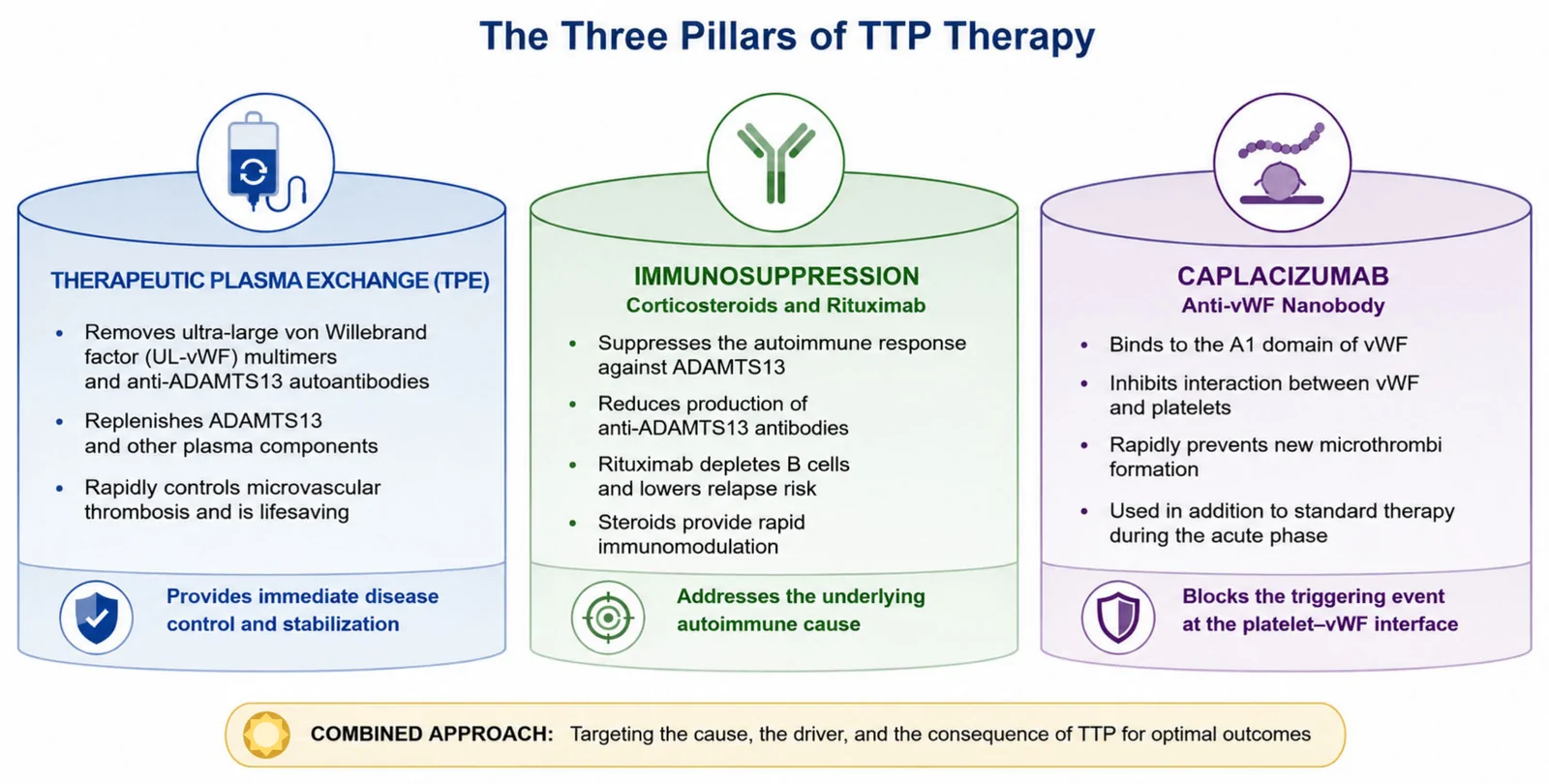
The three complementary therapeutic principles in iTTP are plasma exchange, immunosuppression, and caplacizumab.

Over the past decade, however, disease-modifying treatment options have expanded beyond TPE. Corticosteroids and rituximab are now widely used to suppress autoantibody production and promote immunological remission [9]. In parallel, caplacizumab, a bivalent nanobody targeting the A1 domain of VWF, has introduced a targeted anti-adhesive mechanism that directly prevents VWF binding to platelet glycoprotein Ib-IX-V [10,11]. The combination of TPE, immunosuppression, and caplacizumab (Figure 1) has become the modern standard for many patients and is associated with faster disease control, fewer exacerbations, reduced mortality, and a lower TPE burden [7,10,11].

The availability of caplacizumab has also raised a fundamental question: is TPE required as an immediate first-line therapy for every patient with iTTP, or can it be safely deferred or omitted in carefully selected patients? Pharmacological control of the effector pathway with caplacizumab, together with prompt immunosuppression, may be sufficient to achieve early disease control in a subset of patients with non-severe disease [7,12]. This concept has stimulated growing interest in TPE-free strategies, particularly in experienced centers with rapid ADAMTS13 diagnostics and immediate access to rescue TPE.

In this perspective review, we reassess the role of TPE in contemporary iTTP management and propose a framework for individualized, response-adapted decision-making. We do not argue that TPE has become obsolete. Rather, we suggest that its role should be refined: it is indispensable for severe, unstable, or refractory disease, but potentially avoidable as routine first-line therapy in selected patients under strictly defined conditions.

## 2. Risks and Limitations of Therapeutic Plasma Exchange

TPE is an invasive, technically demanding, and resource-intensive procedure. It often requires central venous catheter (CVC) placement, specialized apheresis infrastructure, trained staff, plasma products, and close monitoring. In many patients, it contributes to intensive care unit utilization and prolongs hospitalization. These practical limitations are particularly relevant in smaller hospitals, during periods of plasma shortage, and in patients in whom vascular access is difficult or contraindicated.

Procedure-related complications include hemodynamic instability due to rapid fluid shifts, citrate-associated hypocalcemia, allergic reactions to donor plasma, and disturbances of coagulation factors and other protective plasma components [13]. CVC placement introduces additional risks, including bleeding, thrombosis, pneumothorax, local infection, and central line-associated bloodstream infection [13,14,15]. Plasma-related reactions range from mild urticaria to severe anaphylaxis [13]. Although these risks have historically been accepted because TPE is life-saving, their relative weight should be reconsidered when rapid disease control is achievable with less invasive therapy in selected patients.

TPE also has mechanistic limitations. Although it removes anti-ADAMTS13 autoantibodies and provides functional ADAMTS13, it does not suppress ongoing autoantibody production. In addition, TPE does not directly block the interaction between VWF and platelets, which is the immediate effector mechanism of microvascular thrombosis in iTTP [16]. Repeated procedures may reduce antibody burden, but durable remission depends on controlling the underlying autoimmune process [17]. These limitations underscore the importance of integrating TPE with immunosuppressive and anti-VWF therapies and support a critical reassessment of whether TPE is required in all patients from the outset.

## 3. Caplacizumab and Integration with Immunosuppressive Therapy

Caplacizumab binds the A1 domain of VWF and prevents its interaction with the platelet glycoprotein Ib-IX-V receptor. By interrupting VWF-mediated platelet adhesion, it acts directly at the final common pathway of iTTP-associated microvascular thrombosis [10]. Preclinical data in a baboon model of acquired TTP provided early support for this mechanism [18], and randomized trials subsequently demonstrated clinical benefit when caplacizumab was added to TPE-based standard therapy [10,19].

In the TITAN and Hercules trials, caplacizumab accelerated platelet count normalization and reduced exacerbations compared with placebo when used in addition to TPE and immunosuppression [10,19,20]. Real-world cohorts have confirmed that early caplacizumab initiation is associated with faster clinical response, fewer recurrences, lower mortality, and a reduced burden of TPE [11,21,22]. These data have led international guidelines to recommend caplacizumab for acute iTTP, both at first presentation and during relapse, with the greatest benefit expected when treatment is started early [7,12,23].

The pharmacological profile of caplacizumab differs fundamentally from that of TPE. Whereas the effects of TPE on autoantibody burden and ADAMTS13 activity accumulate over repeated sessions, caplacizumab can inhibit VWF-platelet interaction rapidly and independently of autoantibody removal or ADAMTS13 replacement [10,11,19,20,21]. This rapid anti-adhesive effect is highly relevant during the acute thrombotic phase. However, caplacizumab does not correct the autoimmune cause of iTTP; durable remission requires immunosuppression to eliminate or suppress anti-ADAMTS13 autoantibody production [24].

Corticosteroids are typically initiated immediately, and rituximab is increasingly used early to accelerate immunological remission and reduce relapse risk [6,7,24,25]. Emerging immunomodulatory approaches, including alternative anti-CD20 antibodies such as obinutuzumab, plasma cell-targeted therapy with daratumumab, bispecific antibodies, and cellular therapies, are being explored for refractory or relapsing disease [26,27,28,29,30]. Additional strategies, such as modified ADAMTS13 variants, recombinant ADAMTS13-based approaches, gene therapy, and platelet-directed ADAMTS13 delivery, may further reshape treatment in the future [31,32,33,34].

Together, these developments have shifted the therapeutic paradigm. If caplacizumab rapidly controls the thrombotic effector mechanism and immunosuppression controls autoantibody production, the obligatory role of immediate TPE for every patient becomes biologically and clinically less certain. This does not diminish the importance of TPE, but it supports a more selective and response-adapted use.

## 4. Evidence for TPE-Free Treatment Strategies

Initial evidence for TPE-free iTTP treatment emerged from exceptional clinical circumstances, including refusal of blood products, inability to obtain central venous access, severe plasma reactions, or pandemic-related limitations of apheresis capacity [35,36,37,38,39,40]. Although these early reports were limited by small sample sizes and selection bias, they demonstrated that clinical remission without TPE is possible when caplacizumab and immunosuppression are initiated promptly.

Subsequent registry-based and cohort data from experienced centers provided more systematic support. Kühne et al. compared patients treated without additional TPE (n = 42) with patients treated with TPE-based therapy (n = 59) and found no significant difference in median time to platelet count normalization, clinical response, or exacerbation rates [12]. These data suggest that caplacizumab plus immunosuppression may be sufficient in selected patients, provided that close monitoring and immediate access to rescue TPE are ensured.

Systematic reviews have reached similar conclusions, indicating that TPE-free treatment can be effective and feasible in selected patients with first-episode, relapsed, or refractory iTTP [41,42]. Nevertheless, the available literature remains heterogeneous, and many reports are observational, retrospective, or limited to highly specialized centers. Therefore, TPE-free strategies should not be generalized indiscriminately to all patients with suspected or confirmed iTTP.

The phase III MAYARI trial was designed to evaluate caplacizumab plus immunosuppression without first-line TPE in adults with iTTP [43,44]. Results presented as a late-breaking abstract at the 2025 ISTH Congress reported remission without TPE in 44 of 46 patients (95.7%); two patients required TPE [44]. These results are encouraging, but they must be interpreted in light of the single-arm design, defined eligibility criteria, and the need for full peer-reviewed publication. They support the feasibility of TPE-free treatment in carefully selected patients, but do not eliminate the need for TPE in patients with severe, unstable, or refractory disease.

Taken together, the current evidence challenges the concept that TPE must be mandatory for all patients as the first therapeutic step. It also reinforces the need for strict patient selection, rapid diagnostic confirmation, structured monitoring, and predefined criteria for escalation to TPE.

## 5. Patient Selection and Clinical Decision-Making

The current standard of care for iTTP therapy consists of plasma exchange, immunosuppression, and caplacizumab. The key clinical challenge is to identify patients in whom TPE can be safely deferred without increasing the risk of irreversible organ injury or death. This is difficult because iTTP may deteriorate rapidly, and initial symptoms do not always predict subsequent severity [45]. Existing clinical scores can support diagnostic probability assessment, but no score has sufficient sensitivity and specificity to reliably determine whether TPE can be omitted in an individual patient [45,46]. Therefore, TPE-free treatment should be considered only in patients who are clinically stable, have no evidence of severe organ involvement, and can be monitored intensively in a center with immediate TPE availability. The immediate availability and prompt initiation of caplacizumab, together with the capacity to initiate rescue TPE without delay if required, are essential prerequisites for any TPE-free treatment strategy.

Previous observational studies suggest that relapsed iTTP episodes may present with less severe clinical and laboratory abnormalities than first acute episodes [47,48]. However, relapse status alone should not be considered sufficient justification for omitting TPE, as this association may be influenced by earlier recognition and treatment, survivor bias, and patient selection, and severe or fatal relapses remain possible. The eligibility criteria of the MAYARI trial provide a useful, although not definitive, framework. Patients were eligible if they had a French thrombotic microangiopathy score of 1 or 2, with iTTP confirmed by ADAMTS13 activity testing within 2 days, and relatively preserved renal function [49]. Exclusion criteria included alternative thrombotic microangiopathies such as atypical hemolytic uremic syndrome, thrombocytopenia from other causes, inherited or acquired coagulation disorders, malignant arterial hypertension, severe neurological or cardiac disease, and the need for chronic anticoagulant or antiplatelet therapy that could not be safely interrupted [49].

**Figure 2:**
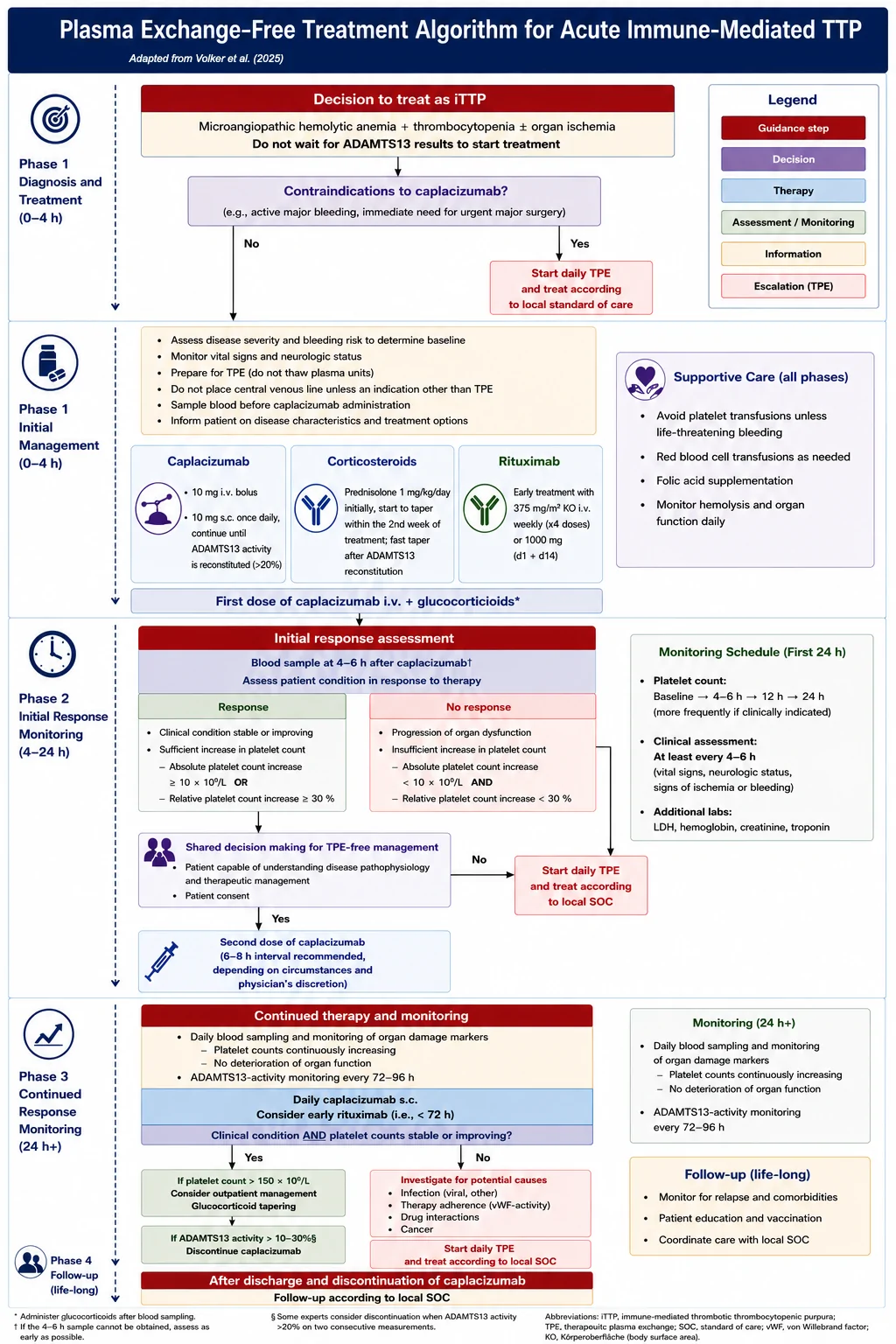
Plasma exchange-free treatment algorithm for acute iTTP based on immediate caplacizumab, immunosuppression, supportive care, close monitoring, and predefined escalation criteria [45]. Created by the authors based on the current literature, conceptually informed by Kühne et al. [12]. Proposed by the authors as a conceptual framework; this algorithm does not constitute an official guideline or formal treatment recommendation.

A practical approach proposed by Völker and Brinkkoetter emphasizes rapid ADAMTS13 testing, immediate initiation of corticosteroids, caplacizumab, and rituximab once iTTP is established or highly likely, as well as maintenance of TPE as a readily available rescue option [45]. Rapid on-site or collaborative ADAMTS13 testing, ideally within hours, is central to this strategy. If platelet counts stabilize or increase and clinical parameters improve after caplacizumab initiation, TPE may be deferred. Conversely, neurological deterioration, cardiac involvement, progressive hemolysis, falling platelet counts, hemodynamic instability, diagnostic uncertainty, or lack of early response should prompt immediate escalation to TPE (Figure 2).

## 6. Monitoring During TPE-Free Treatment

Close monitoring is essential, regardless of whether TPE is used. During the first 24–48 h after caplacizumab initiation, complete blood counts, hemolysis markers, renal function, cardiac markers (when indicated), and neurological status should be assessed frequently [12,45]. Platelet recovery is an important early marker of clinical response, but platelet count alone does not fully reflect autoimmune disease activity.

Serial ADAMTS13 activity monitoring provides a more direct assessment of the underlying disease process and should guide the duration of caplacizumab and immunosuppression [45,50]. Persistent severe ADAMTS13 deficiency at the time of caplacizumab discontinuation is associated with an increased risk of unfavorable outcomes, including exacerbation, relapse, or death [51]. In contrast, stopping caplacizumab after ADAMTS13 activity has recovered above critical thresholds appears to reduce the risk of exacerbation [51].

An ADAMTS13 activity level above 20% is commonly interpreted as evidence of partial immunological remission and meaningful control of the autoimmune process [7,45]. Because recovery of ADAMTS13 activity often takes several weeks, caplacizumab and monitoring generally must be continued beyond platelet count normalization until the underlying disease is adequately controlled [12,45,52]. This is particularly important in TPE-free strategies, where clinical improvement may occur before immunological remission is achieved.

Implementation also requires robust interdisciplinary coordination. Hematology, nephrology, transfusion medicine, intensive care, emergency medicine, and laboratory services must agree on diagnostic pathways, drug availability, monitoring schedules, and escalation criteria. Caplacizumab must be available immediately, and TPE must remain logistically ready for patients who fail to respond or who deteriorate. Without these prerequisites, TPE-free treatment should not be attempted outside clinical trials or specialized protocols.

## 7. Open Questions and Future Directions

Several questions remain unresolved. First, validated criteria are needed to identify patients who can safely start treatment without TPE. Second, the optimal timing, intensity, and duration of immunosuppression in TPE-free strategies must be defined. Third, ADAMTS13-guided stopping rules for caplacizumab require further standardization. Fourth, future studies should determine whether TPE-free approaches reduce catheter-related complications, plasma exposure, hospital length of stay, costs, and patient burden without compromising survival or increasing relapse risk.

Prospective comparative trials and high-quality registry studies are needed to confirm the safety of TPE-free treatment beyond highly selected populations and expert centers. Long-term outcomes, including relapse, neurocognitive sequelae, cardiovascular events, health-related quality of life, and immunological remission, should be incorporated into future studies. Until such data are available, TPE-free treatment should be implemented cautiously, with transparent documentation of eligibility, response, rescue criteria, and outcomes.

Until more data are available, patients with severe neurological or cardiac involvement, malignant hypertension, clinically significant bleeding, substantial renal impairment, or an immediate need for invasive procedures or organ support should receive the standard of care, including TPE as first-line treatment.

## 8. Limitations

This perspective has several limitations. The evidence supporting TPE-free treatment is still based predominantly on case reports, retrospective observational cohorts, and highly selected patients managed at expert centers, while prospective randomized comparisons with TPE-based therapy are lacking, and the MAYARI results have so far been reported only from a single-arm study.

This perspective focuses on adult patients with iTTP. Pediatric iTTP was not specifically evaluated, and the available evidence is insufficient to assess the safety or efficacy of TPE-free treatment in children; consequently, the conclusions and proposed algorithm should not be extrapolated to pediatric patients.

Moreover, as this is a narrative perspective rather than a systematic review, selection and interpretation of the available evidence may be subject to bias; therefore, the proposed patient selection and escalation criteria should be considered hypothesis-generating and should not yet be generalized to routine care outside experienced centers or prospective protocols.

## 9. Conclusions

TPE remains a life-saving treatment for iTTP and should remain immediately available for patients with severe, unstable, refractory, or diagnostically uncertain disease. However, caplacizumab and modern immunosuppression have changed the therapeutic context in which TPE is used. In selected, clinically stable patients managed in experienced centers with rapid ADAMTS13 testing, immediate access to caplacizumab and rituximab, and predefined criteria for rescue TPE, omission of routine first-line TPE may be feasible. The future role of TPE in iTTP is therefore likely to be individualized and response-adapted rather than universal. Defining the patients for whom TPE can be safely deferred is now a central priority for clinical research and guideline development.

## Data Availability

No new data were created or analyzed in this study. Data sharing is not applicable to this article.
